# The London Water Supply

**Published:** 1900-08-11

**Authors:** 


### THE LONDON WATER SUPPLY.

The *Lancet* has just issued a Report on the question of the control of the metropolitan water supply. It enters pretty fully into the existing provisions and the various recommendations and suggestions which have been made from time to time by one commission and another, and, finally, it gives its own conclusions, which are to the following effect: That the auditor appointed by the Local Government Board should be in all respects an officer of that Board, that his salary should be paid from public sources, and that he should be able to obtain the advice and help of the law officers of the Crown; that the Water Examiner appointed by the Local Government Board should have access to all parts of the metropolitan water companies' undertakings. He should be entirely responsible for the report which he issues monthly. The companies should be obliged to place at his disposal every facility for making the several investigations which he wishes. Every investigation which the Water Examiner makes should be done either by himself or by officers appointed by him, and any analyst whom he may employ should be nominated by himself, and the work necessary to enable him to give full and accurate information should be paid for by the Government department. There is surely not a single one of these recommendations to which any fair-minded man could take exception, although it may not be very apparent where they touch the question of control. They seem chiefly to touch the problem of getting information as to what is going on within the works of the various companies, and we may say at once that the chief utility of this report lies not in its recommendations, but in the fact that by reading "between the lines," as the saying goes, one sees how helpless the London consumer, as represented by the various sanitary authorities headed by the London County Council, is in the matter.

If we take the suggested reforms as indicating in each case that there exists something to reform, we find that the Government auditor is paid by the companies, that the Water Examiner appointed by the Government to report on the condition of the water has not access to all parts of water works, that he is unable to obtain all the facilities which are required for making his investigations, that the investigations professing to be made by the Water Examiner are not done by himself or by officers appointed by him, and, lastly, that when the Water Examiner requires any analysis to be made this is not done by people nominated by himself or paid for by the Government. If this is a true description of the sort of supervision over the water supply of the metropolis which is permitted by law at the present time things are evidently in a pretty bad way, and that they are so is, we think, a fair deduction from the recommendations put forward by the *Lancet* Commission. What seems clear is that it is only by the courtesy of the companies that we know anything about what is going on inside their works; that if they want to hide anything, all they have to do is to shut the door; and that many of the reports on the conditions of the water which are published in Blue Books are really prepared and given to the Government department by officials appointed and paid by the companies! Hardly a business-like arrangement.

